# Supplementary material for: Site-Directed Mutagenesis Mediated by Molecular Modeling and Docking and Its Effect on the Protein–Protein Interactions of the bHLH Transcription Factors SPATULA, HECATE1, and INDEHISCENT
Source: Plants (Basel). 2025 Jun 8;14(12):1756. doi: 10.3390/plants14121756 (PMC12196928; doi:10.3390/plants14121756)
Supplement: Supplementary file 1 [file plants-14-01756-s001.zip › plants-3625220-supplementary.pdf]

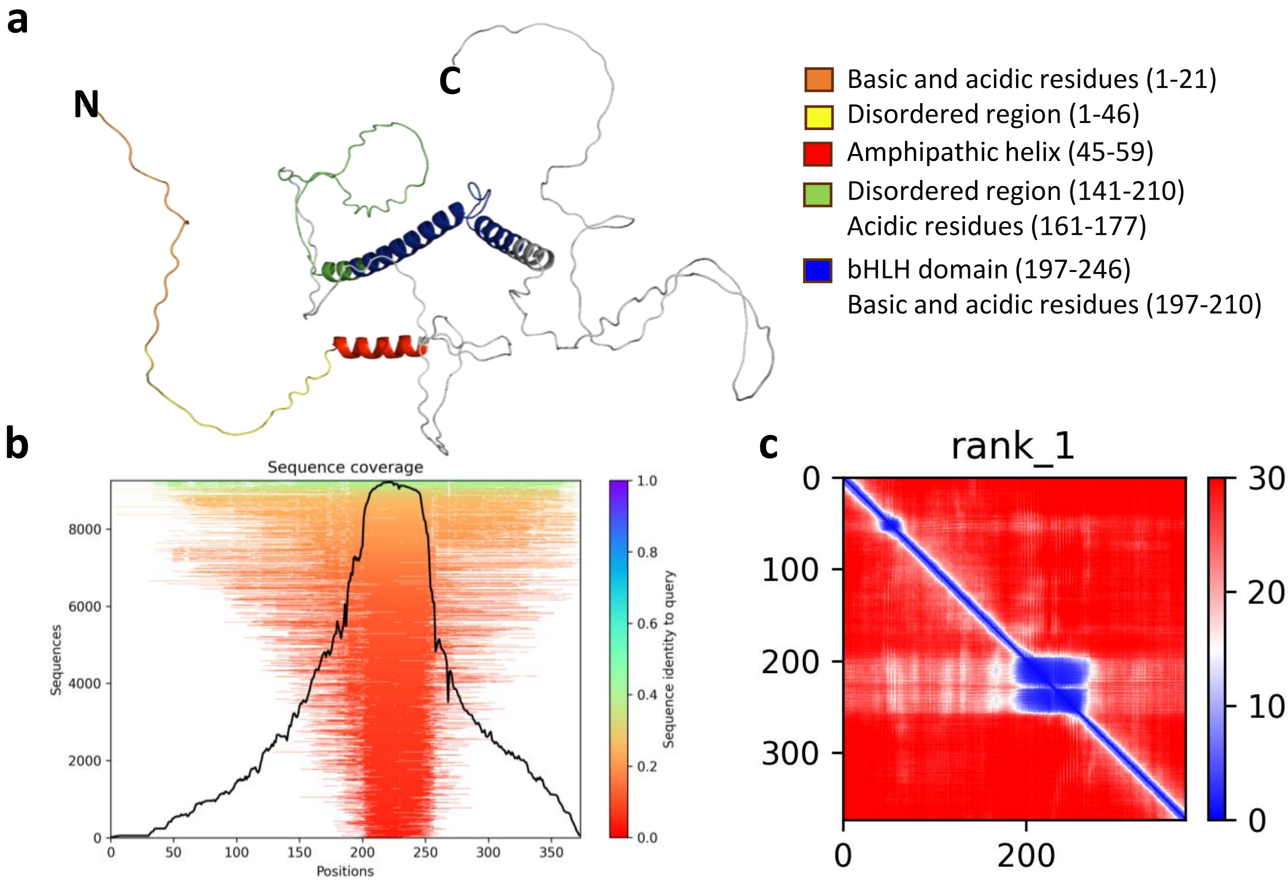

Figure S1. Results of the protein modeling of SPATULA (SPT) with AlphaFold2. **(a)** Description of the three-dimensional structure of SPT, based on the described UniProt families and domains. N- and C-termini are labeled. **(b)** SPT sequence coverage against a little more than 8000 sequences that were used to build the modeled SPT structure. **(c)** Visualization of the predicted alignment errors in Ångström (Å), in blue are shown the sequence with the highest prediction confidence corresponding to the region between 190 to 260 residues approximately.

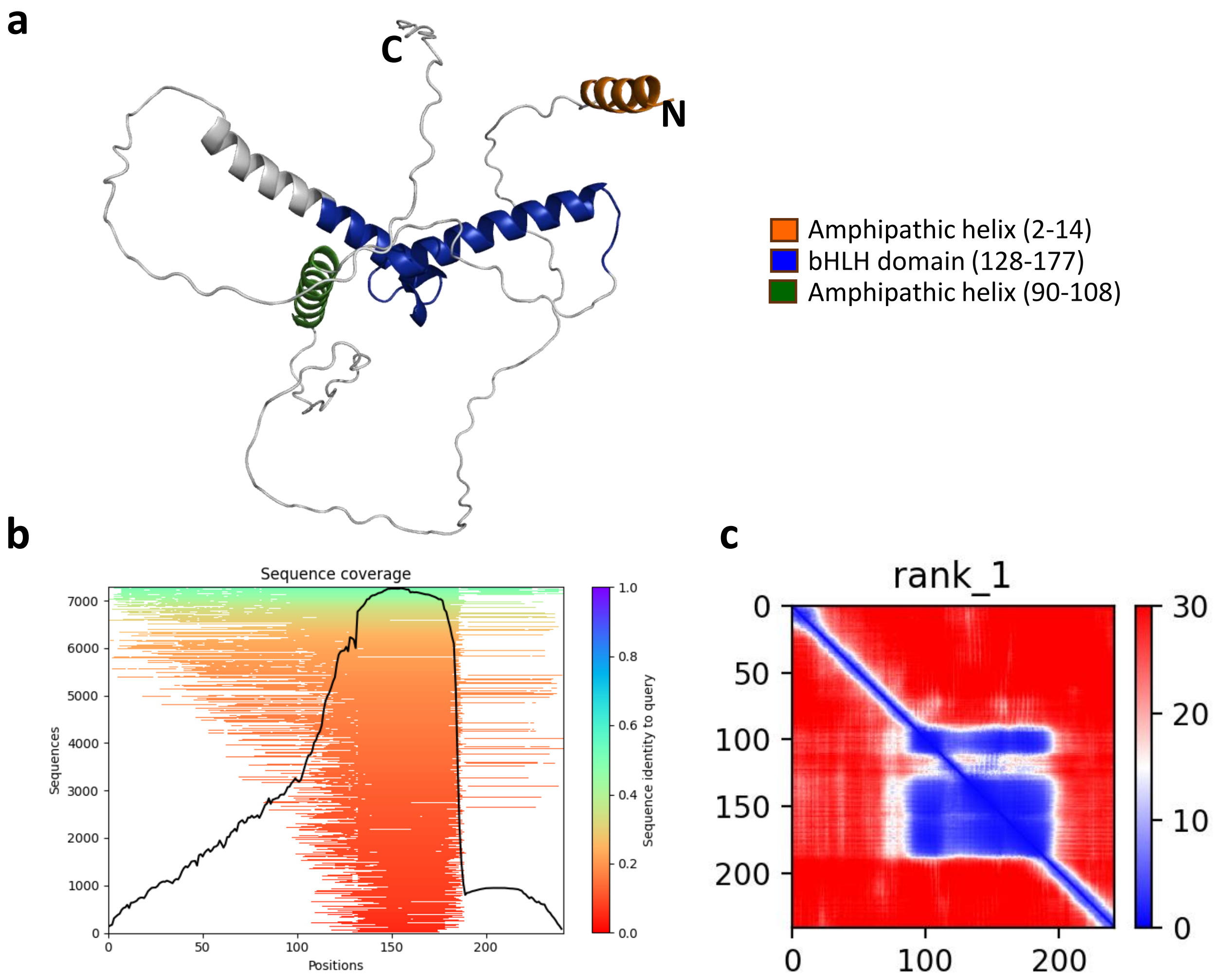

Figure S2. Results of the protein modeling of HECATE1 (HEC1) with AlphaFold2. **(a)** Description of the three-dimensional structure of HEC1, based on the described UniProt families and domains. N- and C-termini are labeled. **(b)** HEC1 sequence coverage against a little more than 7000 sequences that were used to build the modeled structure of HEC1. **(c)** Visualization of the predicted alignment errors in Ångström (Å), in blue are shown the sequence with the highest prediction confidence corresponding to the regions between residue 95 to 120 and 130 to 180 approximately.

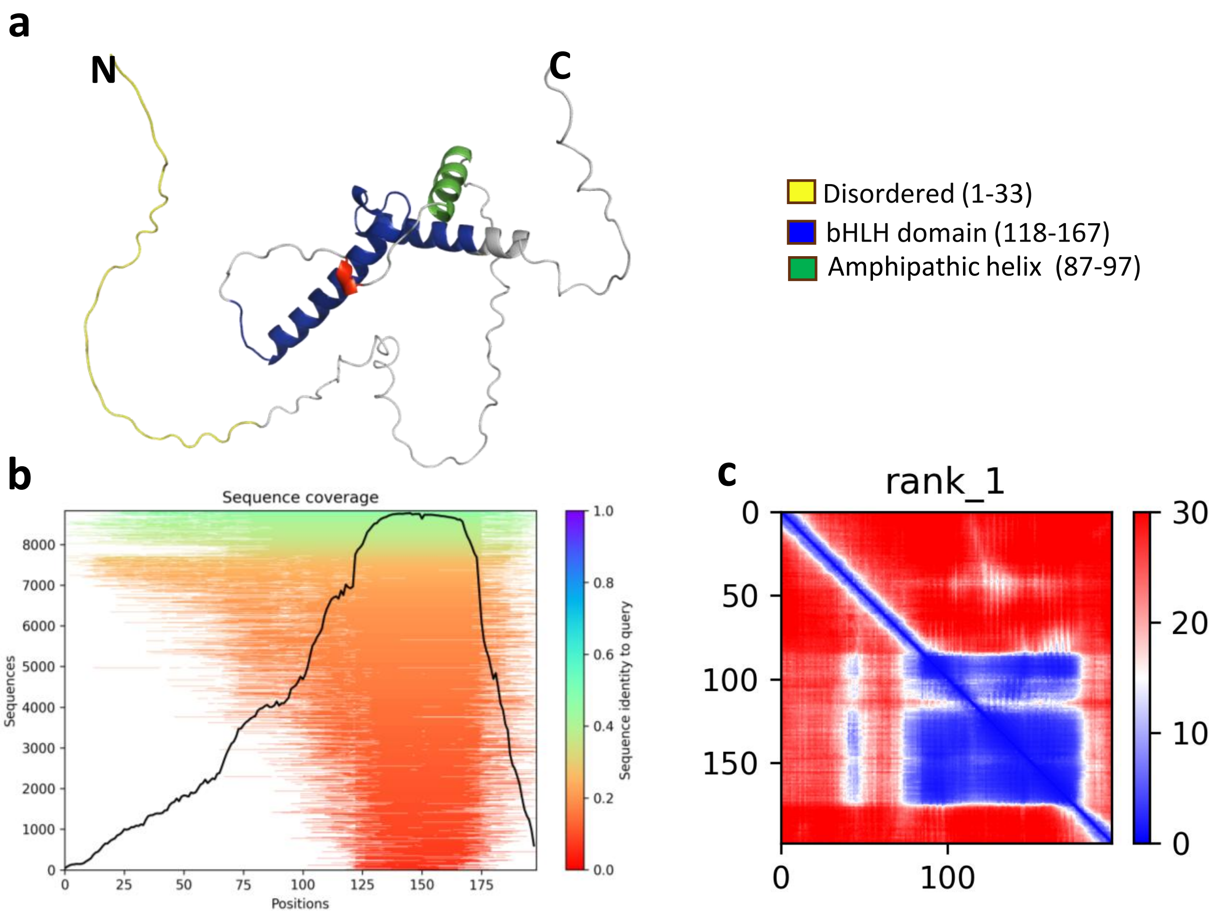

Figure S3. Results of the protein modeling of INDEHISCENT (IND) with AlphaFold2. **(a)** Description of the three-dimensional structure of IND, based on the described families and domains of UniProt. N- and C-termini are labeled. **(b)** IND sequence coverage against a little more than 8000 sequences that were used to build the modeled IND structure. **(c)** Visualization of the predicted alignment errors in Ångström (Å), in blue are shown the sequence with the highest prediction confidence that corresponds to the region between residue 85 to 180 approximately.

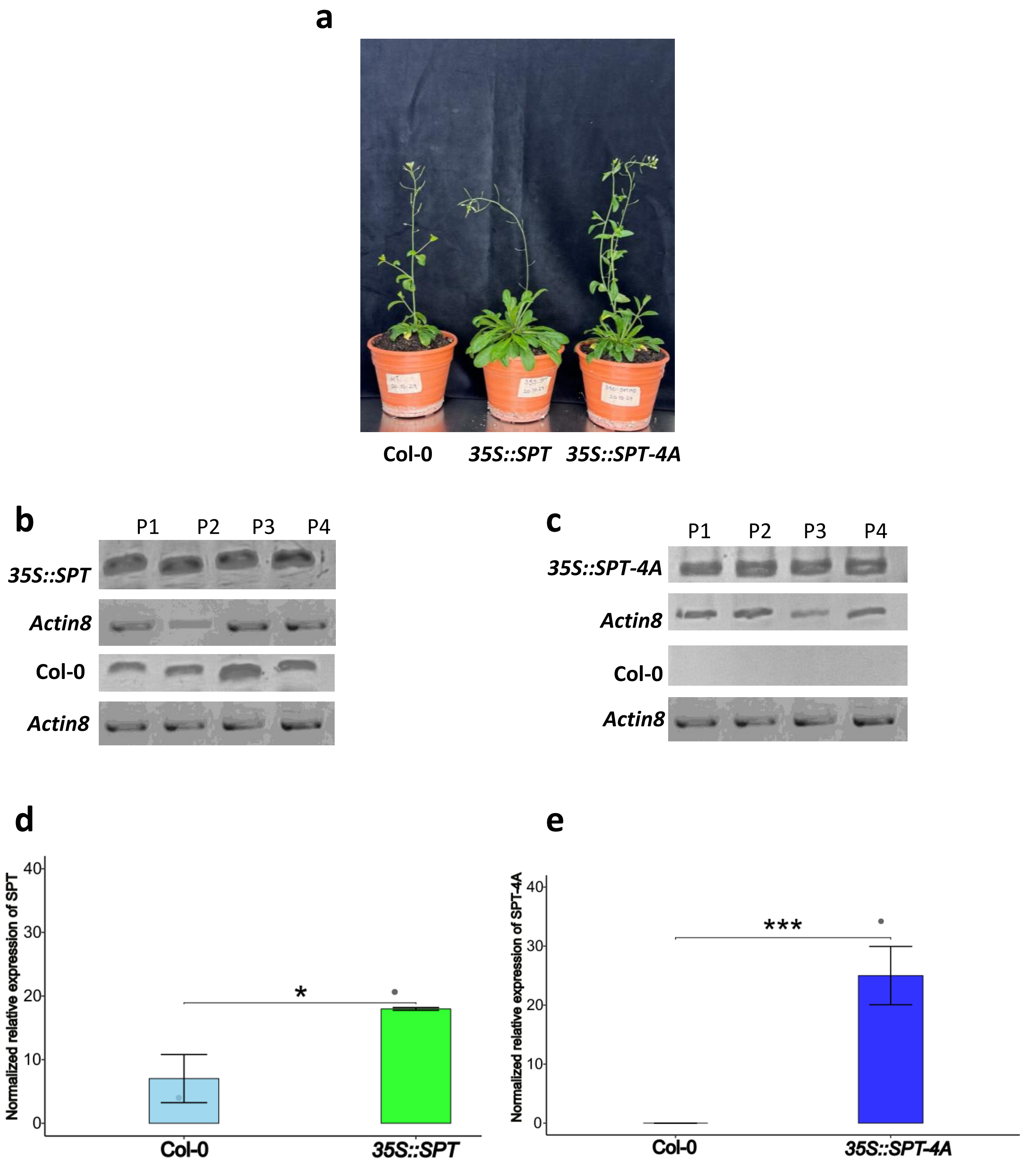

Figure S4. *SPT* overexpression Arabidopsis plants. **(a)** Plants of the wild-type (Col-0), the *35S::SPT* and the *35S::SPT-4A* lines. **(b, d)** Expression levels of the SPT allele detected by RT-PCR in the *35S::SPT* and wild-type (Col-0). **(c, e)** Expression levels of the *SPT-4A* allele revealed by RT-PCR in the *35S::SPT-4A* and wild-type (Col-0). Statistical analyses were performed using a Wilcoxon test $n = 4$, *$p < 0.05$, ***$p < 0.001$, ns $p > 0.05$. Note: images/measurements of wild-type (Col-0) for actin amplification are the same as in Figures S5 and S6.

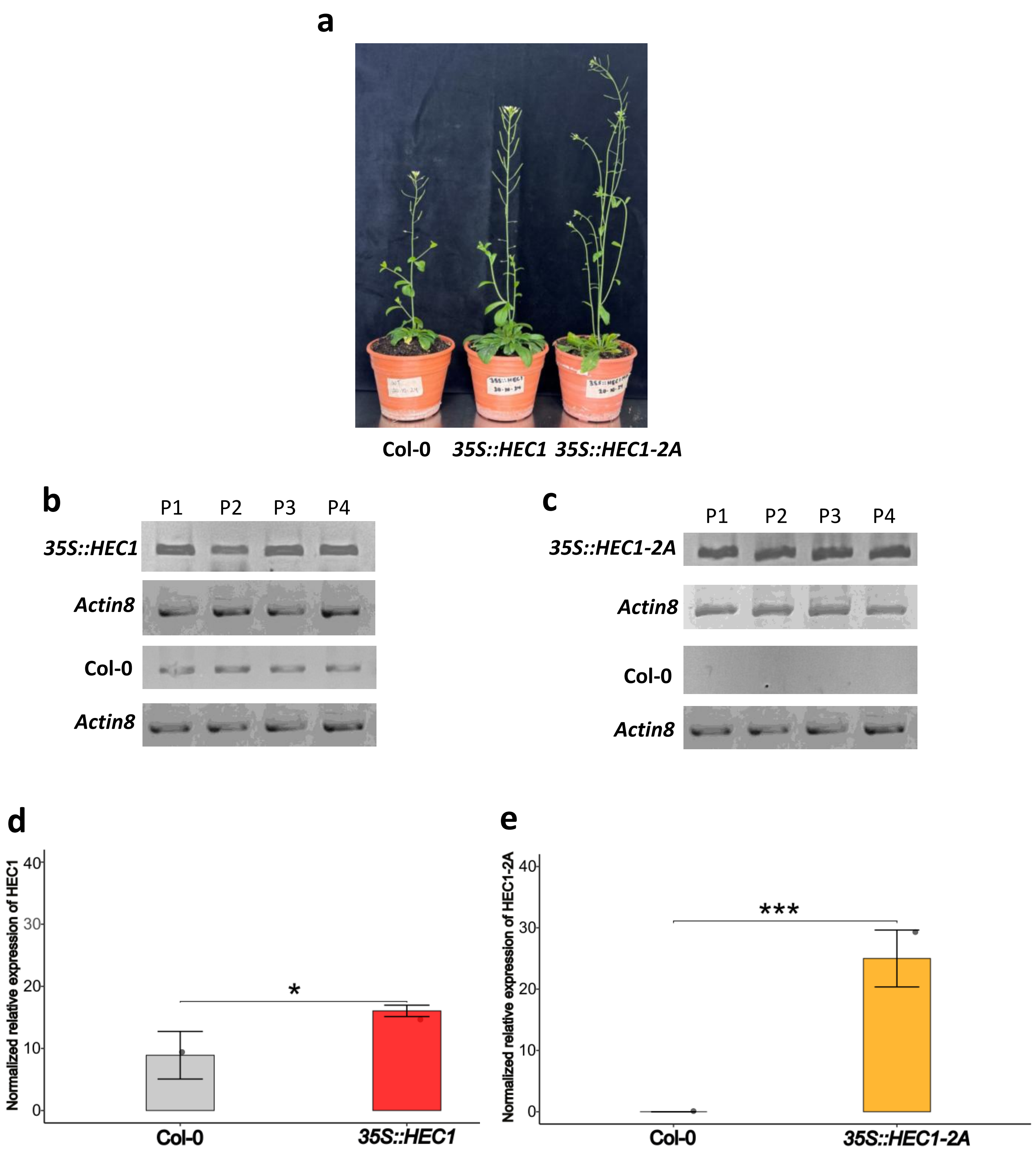

Figure S5. *HEC1* overexpression Arabidopsis plants. **(a)** Plants of the wild-type (Col-0), the *35S::HEC1* and the *35S::HEC1-2A* lines. **(b, d)** Expression levels of the *HEC1* allele in the *35S::HEC1* and wild-type (Col-0). **(c, e)** Expression levels of the *HEC1-2A* allele revealed by RT-PCR in the *35S::HEC1-2A* and wild-type (Col-0). Statistical analyses were performed using a Wilcoxon test $n = 4$, $^{*}p < 0.05$, $^{***}p < 0.001$, ns $p > 0.05$. Note: images/measurements of wild-type (Col-0) for actin amplification are the same as in Figures S4 and S6.

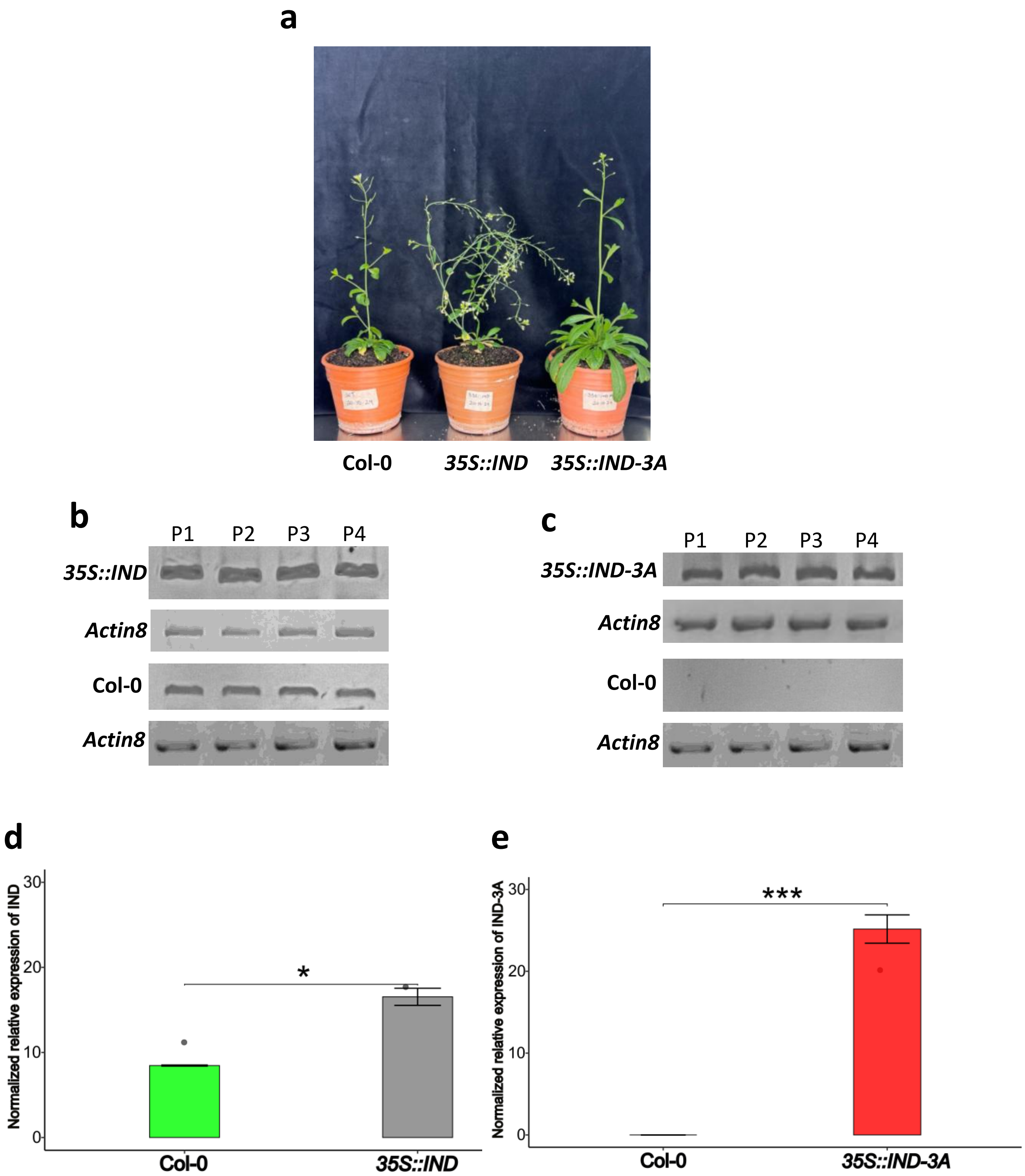

Figure S6. *IND* overexpression Arabidopsis plants. **(a)** Plants of the wild-type (Col-0), the *35S::IND* and the *35S::IND-3A* lines. **(b, d)** Expression levels of the *IND* allele in the *35S::IND* and wild-type (Col-0). **(c, e)** Expression levels of the *IND-3A* allele revealed by RT-PCR in the *35S::IND-3A* and wild-type (Col-0). Statistical analyses were performed using a Wilcoxon test n = 4, *$p < 0.05$, ***$p < 0.001$, ns $p > 0.05$. Note: mages/measurements of wild-type (Col-0) for actin amplification are the same as in Figures S4 and S5.

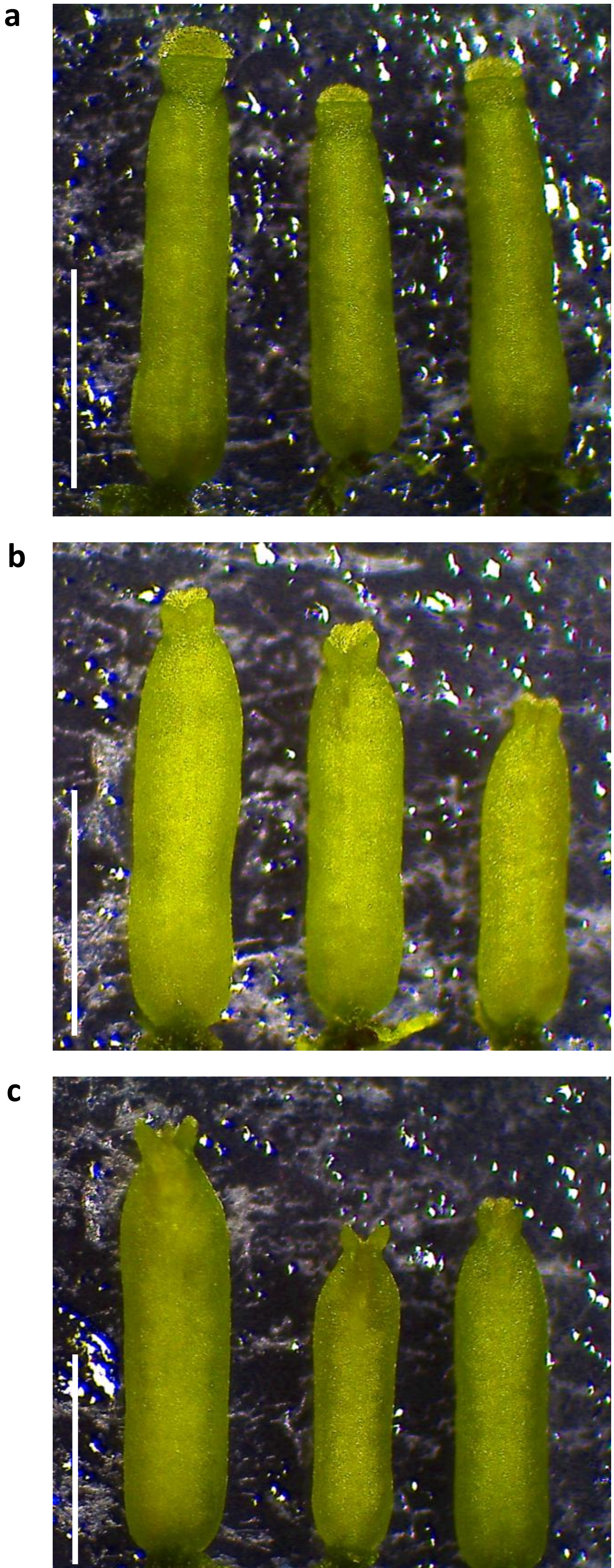

Figure S7. Gynoecia phenotypes. **(a)** Gynoecia phenotypes of wild-type (Col-0), **(b)** *35S::SPT-4A,* and **(c)** *spt-12.* Scale bars = 1 mm.

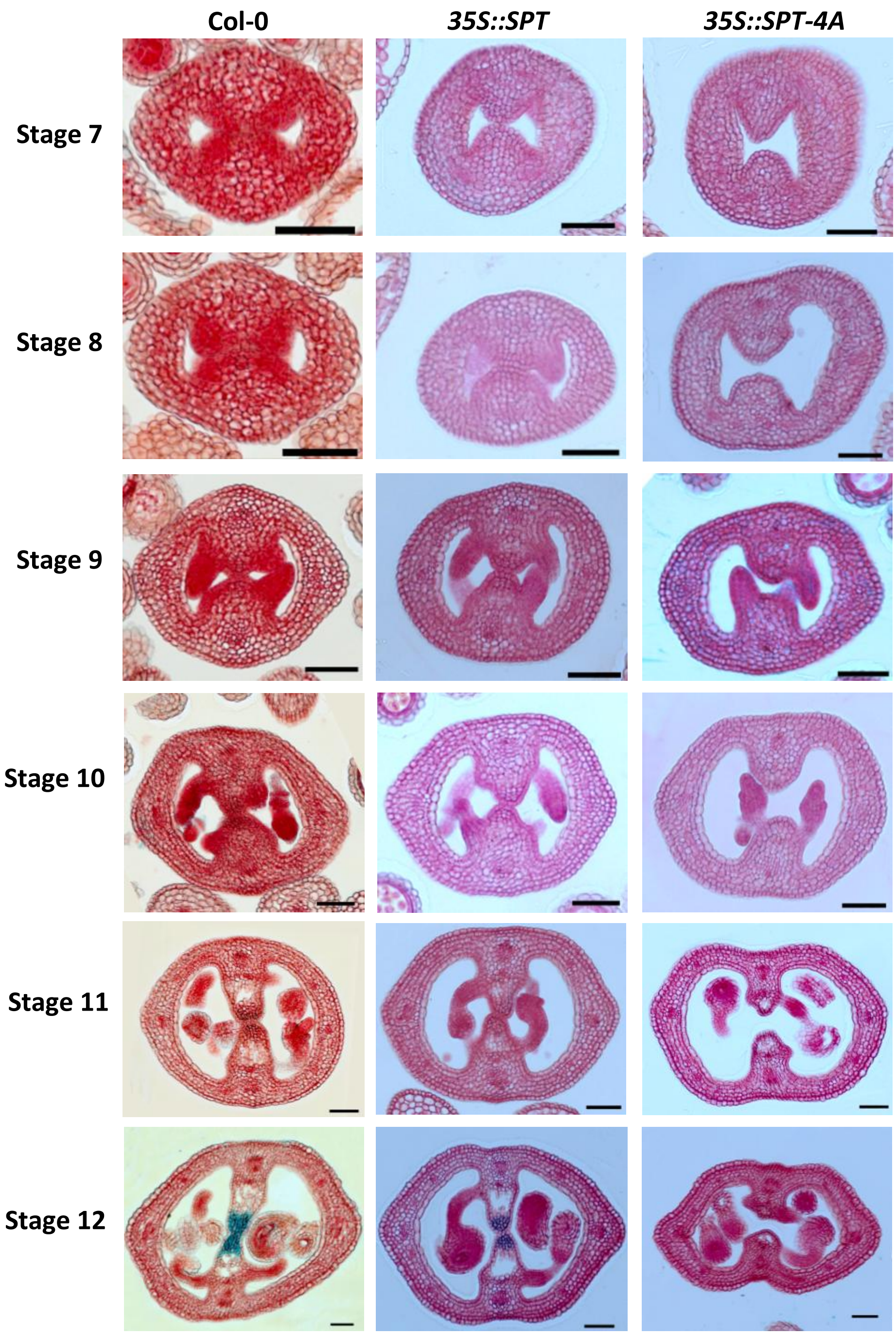

Figure S8. Gynoecia cross-sections stained with alcian blue and neutral red staining of wild-type (Col-0), *35S::SPT* and *35S::SPT-4A* lines at stage 7 to 12. Images were digitally extracted for comparison. Note: images/measurements of wild-type (Col-0) samples are the same as in Figures S9 and S10. Scale bars = 50 µm.

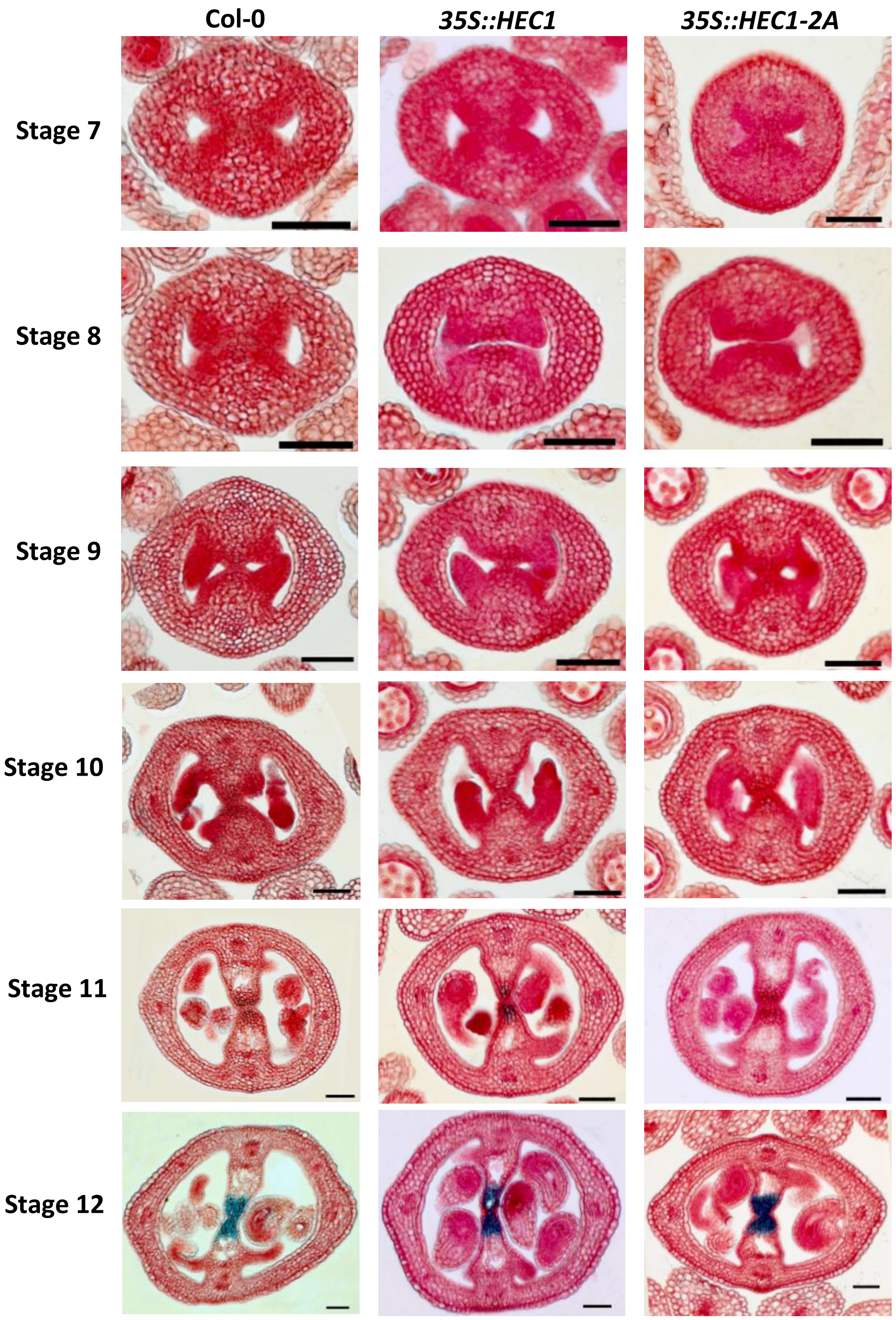

Figure S9. Gynoecia cross-sections stained with alcian blue and neutral red staining of wild-type (Col-0), *35S::HEC1* and *35S::HEC1-2A* lines at stage 7 to 12. Images were digitally extracted for comparison. Note: images/measurements of wild-type (Col-0) samples are the same as in Figures S8 and S10. Scale bars = 50 µm.

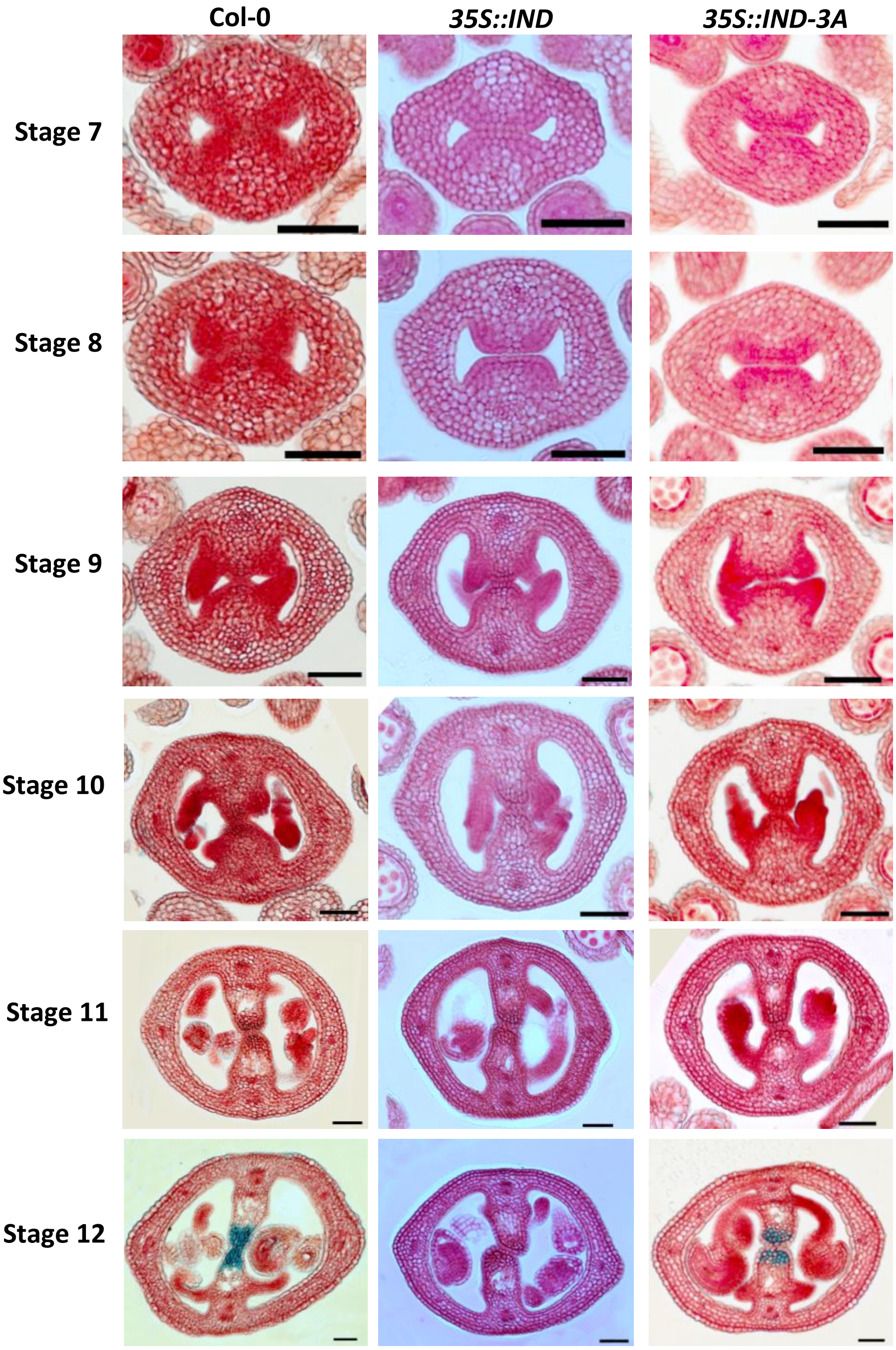

Figure S10. Gynoecia cross-sections stained with alcian blue and neutral red staining of wild-type (Col-0), *35S::IND* and *35S::IND-3A* lines at stage 7 to 12. Images were digitally extracted for comparison. Note: images/measurements of wild-type (Col-0) samples are the same as in Figures S8 and S9. Scale bars = 50 µm.

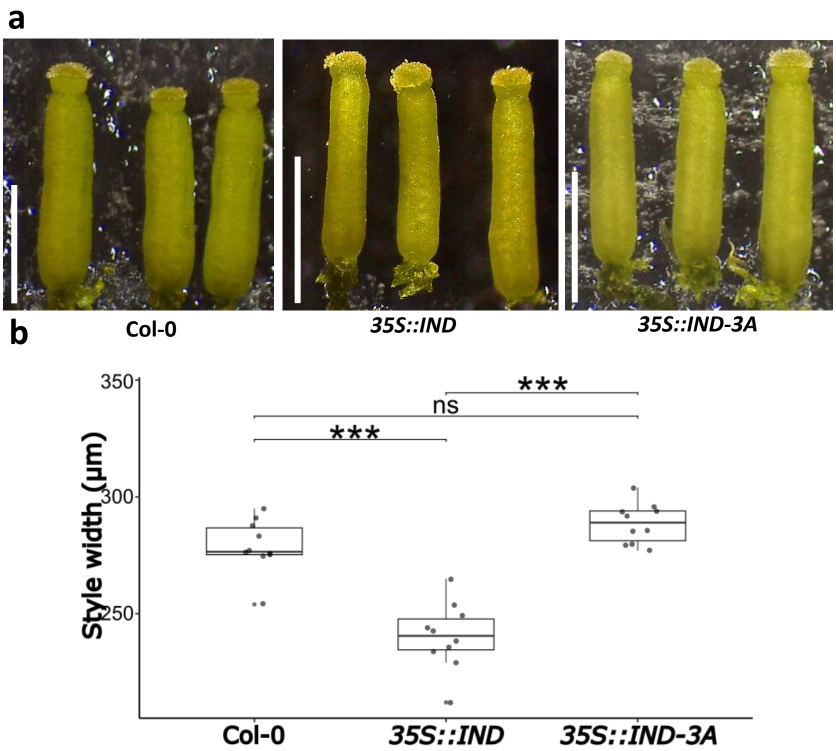

Figura S11. Functional analysis of *35S::IND* and *35S::IND-3A* gynoecia. **(a)** Gynoecium of wild-type (Col-0), *35S::IND* and *35S::IND-3A*. **(b)** Quantitative analyses of style width of gynoecium of wild-type (Col-0), *35S::IND* and *35S::IND-3A*. ANOVA followed by Tukey´s test, ***$p < 0.001$, **$p < 0.01$, *$p < 0.05$, ns ≥ 0.05, $n = 10$. Note: images/measurements of wild-type (Col-0) samples are the same as in Figure 7. Scale bars = 1 mm.

VN:SPT VC:empty

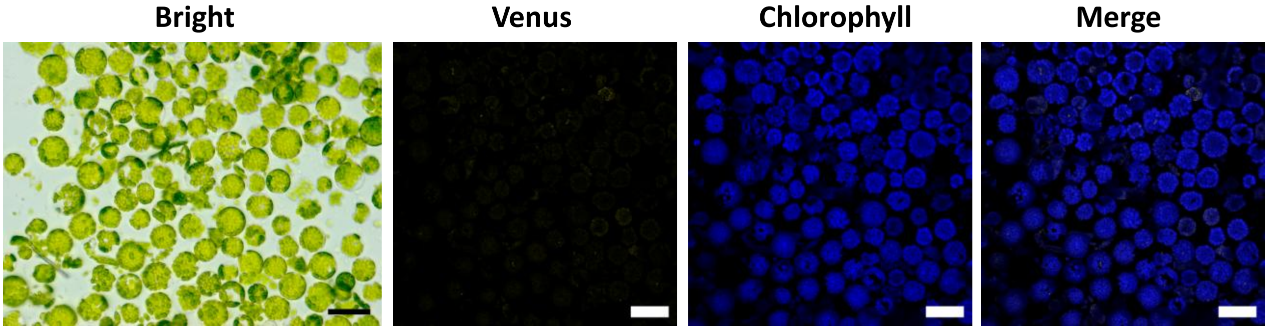

Figure S12. Negative control for the BiFC assay with a 20X objective. No fluorescence signal is observed in protoplasts with the combination *VN:SPT VC:empty*. Scale bars = 50 μm.

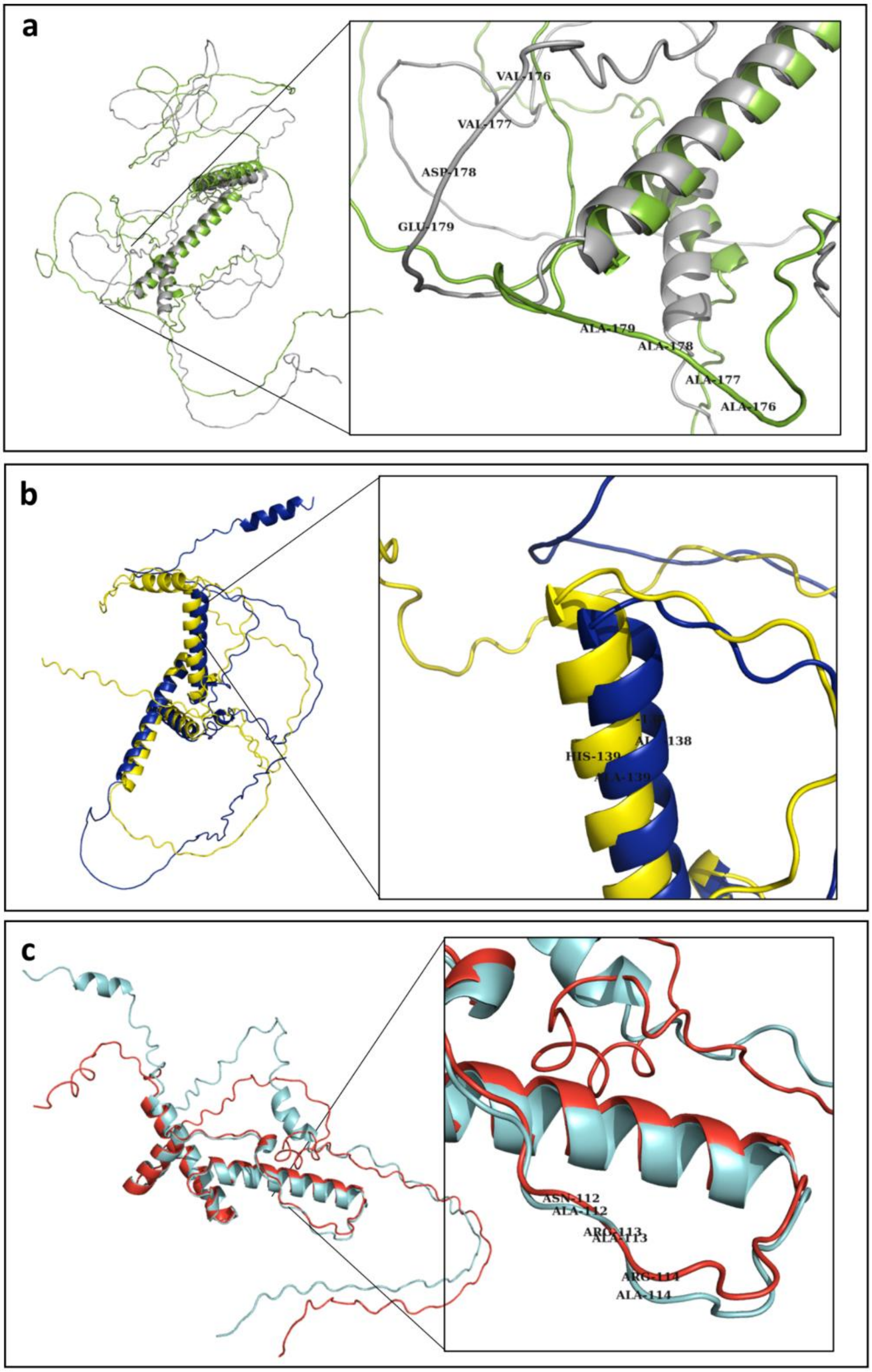

Figure S13. Comparison of the modeled structures of the studied proteins. **(a)** Modeled structure of SPT (gray) and SPT-4A (green). **(b)** Modeled structure of HEC1 (yellow) and HEC1-2A (blue). **(c)** Modeled structure of IND (red) and IND-3A (cyan). The modeled structures were generated in AlphaFold2, prepared in Chimera 1.18, and structurally aligned using the PyMOL 3.1.3 plugin.

Table S1. List of primers used for site-directed mutagenesis.

Primer name	Sequence 5´-3´	Number of bases	Description
FW-SDMSPT	GCTGCAGCTCTCCCTTCCAAGTCAGGTCC	29	Forward primer to substitute Val176, Val177, Asp178, and Glu179 for 4x alanine in SPT.
RV-SDMSPT	AGCTGCAGCTTCTCCTCCTTCCCCTCCCGC	30	Reverse primer to substitute Val176, Val177, Asp178 and Glu179 for 4x alanine in SPT.
FW-SDMHEC1	CGTGGCGGCTGCGGGGAGAGAGAG	24	Forward primer to substitute Arg138 and His139 for 2x alanine in HEC1.
RV-SDMHEC1	CTTTGAGGATCTTTAGAGATCC	22	Reverse primer to substitute Arg138 and His139 for 2x alanine in HEC1.
FW-SDMIND	CGCTAACGTAAGGATAAGCGACGATCCTC	29	Forward primer to substitute Asn112, Arg113 and Arg114 for 3x alanine in IND.
RV-SDMIND	GCGGCCGGCTTAGGGACCGTGGCAGGG	27	Reverse primer to substitute Asn112, Arg113 and Arg114 for 3x alanine in IND.

Table S2. List of primers used for RT-PCR.

Primer name	Sequence 5´-3´	Number of bases	Description	Number of bases
FW-SPT	GGAACCTTCTATTCGCAG	18	Forward primer for RT-PCR for *35S::SPT* line.	178
RV-SPT	GGACACTGTTCAAGTAATTCG	21	Reverse primer for RT-PCR for *35S::SPT* line.	
FW-HEC1	CGGATTTTGCAACGGCTTG	19	Forward primer for RT-PCR for *35S::HEC1* line.	282
RV-HEC1	TCATCTAAGAATCTGTGCATTGC	23	Reverse primer for RT-PCR for *35S::HEC1* line.	
FW-IND	GATAAGCGACGATCCTCAGAC	21	Forward primer for RT-PCR for *35S::IND* line.	247
RV-IND	TCAGGGTTGGGAGTTGTG	18	Reverse primer for RT-PCR for *35S::IND* line.	
FW-SDMSPT	GCTGCAGCTCTCCCTTCCAAGTCAGGTCC	29	Forward primer for RT-PCR for *35S::SPT-4A* line.	591
RV-SDMSPT	GGACACTGTTCAAGTAATTCG	21	Reverse primer for RT-PCR for *35S::SPT-4A* line.	
FW-SDMHEC1	CGTGGCGGCTGCGGGGAGAGAGAG	24	Forward primer for RT-PCR for *35S::HEC1-2A* line.	325
RV-SDMHEC1	TCATCTAAGAATCTGTGCATTGC	23	Reverse primer for RT-PCR for *35S::HEC1-2A* line.	
FW-SDMIND	CGCTAACGTAAGGATAAGCGACGATCCTC	29	Forward primer for RT-PCR for *35S::IND-3A* line.	259
RV-SDMIND	TCAGGGTTGGGAGTTGTG	18	Reverse primer for RT-PCR for *35S::IND-3A* line.	

Table S3. Decomposition of binding free energies (MM/GSBA) of the best 10 complex models obtained in the HawkDock platform of the SPT-HEC1 interaction.

Energies	Model1	Model2	Model3	Model4	Model5	Model6	Model7	Model8	Model9	Model10
VDW	-53.93	-51.59	-41.07	-52.9	-60.18	-33.41	-43.49	-33.04	-30.73	-25.22
ELE	-843.58	-989.73	-758.25	-950.03	-420.05	-83.83	-193.61	-227.72	-122.09	-235.89
GB	861.38	1005.58	770.74	965.94	450.93	105.11	216.64	243.95	147.91	245.93
SA	-9.74	-8.01	-7.08	-9.17	-9.06	-4.67	-5.98	-4.67	-4.85	-3.7
TOTAL	**-45.87**	**-43.74**	**-35.66**	**-46.17**	**-38.36**	**-16.8**	**-26.43**	**-21.48**	**-9.76**	**-18.88**

VDW: Van der Waals; ELE: Electrostatic; GB: Generalized Born; SA: Surface Area.

Table S4. Decomposition of binding free energies (MM/GSBA) of the best 10 complex models obtained in the HawkDock platform of the SPT-IND interaction.

Energies	Model1	Model2	Model3	Model4	Model5	Model6	Model7	Model8	Model9	Model10
VDW	-36.69	-43.98	-38.32	-48.23	-36.82	-37.71	-38.99	-60.58	-40.5	-24.86
ELE	-834.16	-529.51	-493.54	-63.25	-843.63	-203.72	-693.13	-255.05	-249.82	-651.36
GB	845.15	537.99	516.14	97.58	849.65	221.89	702.69	287.18	269.69	650.55
SA	-7.16	-8.07	-6.36	-7.13	-7.28	-5.89	-6.98	-9.44	-6.91	-5.02
TOTAL	**-32.85**	**-43.58**	**-22.08**	**-21.03**	**-38.07**	**-25.43**	**-36.41**	**-37.88**	**-27.54**	**-30.69**

VDW: Van der Waals; ELE: Electrostatic; GB: Generalized Born; SA: Surface Area.

Table S5. Summary of the number of independent transformants obtained and scored overexpressing lines.

Lines	Number of independent transformants (T0)	Transformants with phenotype (T1)	Plants used for phenotyping (T2)
35S::HEC1	9	2	10
35S::IND	14	2	10
35S::SPT-4A	14	3	10
35S::HEC1-2A	11	4	10
35S::IND-3A	17	5	10

T2 plants were scored, one plant (the strongest phenotype) from each independent T1 family was selected.
